# To Visit the South

**Published:** 1894-02

**Authors:** 


### TO VISIT THE SOUTH.

### MEDICAL AND SANITARY EXPERTS TO MAKE A TOUR OF INSPECTION.

The Southern Interstate Immigration Bureau has perfected arrangements whereby a party of experienced medical and sanitary experts will make a tour of inspection through the Southern States, and after a careful examination of the territory express their opinion in substantial form.

The object of this inspection, as stated by the bureau, is to correct the erroneous impression that the Southern section of the United States is a territory consisting of swamps and a hotbed for the propagation of contagious and malarial diseases. The medical experts in the party comprise the following: Dr. W. C. Wile, editor of the *New England Medical Monthly*, Danbury, Conn.; Dr. A. N. Bell, editor of the *Sanitarian*, Brooklyn, N. Y.; Dr. H. H. Kynette, editor of the *Medical and Surgical Reporter*, Philadelphia; Dr. W. A. Hammond, Surgeon-General United States Army, retired list, Washington, D. C.; Dr. Ferdinand King, editor of the *Polyclinic*, New York City; Hon. Clark Bell, editor of the *Medical Legal Journal*, New York; T. D. Crothers, editor of the *Journal of Inebriety*, Hartford, Conn.; Dr. T. D. Bailey, editor of the *Brooklyn Medical and Surgical Journal*, Brooklyn, N. Y.; Dr. Howard VanResselaer, editor of the *Medical Annuals*, Albany, N. Y.; Dr. W. Blair Stewart, editor of the *Medical Bulletin*, Philadelphia, Pa.

---

PARTIAL ABLATION OF THE RECTUM.—M. Hartman describes the following operation, which he has successfully performed in cases of annular stenosis of the rectum, either fibrous or neoplastic. The anus having been dilated, the stenosed portion of the rectum is drawn down with a small forceps. A circular incision is then made around the rectum above the stricture and another below the stenosis. The strictured portion of the gut is now excised. The cut section of rectum is then drawn down and sutured to the skin of the anal region. At the end of a week the sutures come away and the rectum retracts, but sufficient fixation has been secured so that suturing is not again required.—*Ex.*

---

CANCER OF RECTUM.—Dr. McCosh makes a posterior or sacral incision, removing the bone part if necessary. If the peritoneum is opened, no attempt is made to close it, but it is packed with odoform gauze. It is claimed, first, that permanent cure may be obtained after repeated incisions; second, that even if the disease returns, life is prolonged more than after colotomy and greater relief afforded. The mortality from operation is about nineteen per cent., permanent cures about eleven per cent., and the operation may be successful even when the disease extends higher than the peritoneal attachment.—*American Doctor.*

